# Elders' Life Stories: Impact on the Next Generation of Health Professionals

**DOI:** 10.1155/2013/493728

**Published:** 2013-08-21

**Authors:** Tracy Chippendale

**Affiliations:** Department of Occupational Therapy, New York University, 35 West 4th Street, 11th Floor, New York, NY 10012, USA

## Abstract

The purpose of this study was to pilot an enhanced version of the “Share your Life Story” life review writing workshop. The enhanced version included the addition of an intergenerational exchange, based on the content of seniors' writings, with students planning careers in the health sciences. The researcher employed a mixed methods design. Preliminary results using descriptive analysis revealed an increase in positive images of aging and a decrease in negative images of aging among the five student participants. Qualitative results revealed six themes that illuminate the hows and whys of the quantitative results as well as additional program benefits. Feedback from students and seniors helped to refine the intergenerational protocol for a larger scale study.

## 1. Introduction

In the United States and in many countries around the globe, the population is aging. The growth of the older adult population raises concerns with regard to the number of health care providers available to care for this population. In a recent report, the Institute of Medicine (IOM) recommended a focused effort on increasing the recruitment and retention of geriatric specialists and caregivers [1]. Many students in medicine and allied health have little interest in working with older adult clients [2]. However, Robert and Mosher-Ashley [3] found that students who had more positive attitudes towards older adults were more likely to be interested in careers in geriatrics. Therefore, interventions that target perceptions of older adults/aging among the younger generation and that promote an interest in working with this population are warranted. Intergenerational programs not only benefit older adults, but can also impact the attitudes of the younger generation [4]. 

 Intergenerational programs can be defined as programs that engage different generations, for example, a younger and older generation, in mutually beneficial planned activities [5]. The majority of studies in this area of inquiry have focused on well elders and primary and secondary students. However, programs that include older adults with cognitive and functional impairments and that involve college students have also been studied [4]. Intergenerational programs can take on a number of forms and incorporate a variety of activities to promote an exchange between the generations. Examples include student run exercise or recreational programs, joint participation in university and college courses, joint participation in civic engagement/volunteer projects, older adult led outdoor classroom activities, reminiscence and life review to name a few [6–9]. Further, intergenerational programming has also been incorporated into medical and allied health education [10, 11]. Documented benefits to the older adult participants include lower stereotypical perceptions of themselves and the student participants, decreased depressive symptoms [7], improved social integration/broader social networks [8], enhanced well being [6], and enjoyment [9]. 

While the intergeneration exchange may benefit older adults, it can also have positive effects on the student participants. Benefits include improved dementia awareness [12], improved school performance, changes in age stereotypes/attitudes towards older adults and changes in attitudes towards aging [7, 10, 13–15]. While a number of programs have been studied and numerous benefits revealed, the current review will now turn towards programs that focus specifically on intergenerational programs that incorporate reminiscence or life review.

Intergenerational reminiscence programs that involve transmission of cultural information to family members and nonrelations have been implemented. Mercken [16] described a program of neighborhood reminiscence in the Netherlands that included the opportunity for seniors from different cultural groups to share life stories with other seniors as well as the younger generation. Exchanges were conducted in the context of the neighborhood environment including schools, playgrounds, and local shops. Although no specific outcome data was reported, the authors concluded that the enjoyment of sharing stories appeared to be universal and that the goals of the program, including improved social cohesion and social integration, were achieved. Adjaye and Aborampah [17] studied intergenerational cultural transmission among the Akan of Ghana. The mechanism by which culture is transmitted includes oral narratives and reminiscences passed down to children by elders. Surveys conducted with 28 cultural group members revealed that respect for elders, civic engagement, and social connectedness were among the important topics for cultural transmission. The researchers concluded that intergenerational transmission of values is a culturally meaningful responsibility and that intergenerational support increased well being. 

Intergenerational reminiscence and life review involving students and seniors have taken a variety of forms. Chung [12] investigated the benefits of a 12-session reminiscence program conducted in eight adult day care centers in Hong Kong using a pretest and posttest one group design. Fifty-one older adults (ages 65 and older) with a diagnosis of dementia and 121 youth participants (ages 16–25) participated in the program. The program included weekly exchanges where youth volunteers acted as facilitators to encourage the older adult participants to share their life experiences. Set reminiscence topics, interactive old-time activities, and reminiscence props were used during the sessions. Youth volunteers also had the opportunity to share their experiences as they related to the reminiscence topics. The results of paired sample *t*-tests revealed a statistically significant improvement in quality of life and depressive symptoms among the older adult participants and a significant change in knowledge of dementia for the youth volunteers. 

Zucchero [18] examined the effects of a biopsychosocial life review conducted in interview format by 67 psychology students paired with older adult volunteers. Students generated an academic paper based on the results of the interviews and wrote personal reflections about their experiences. A poster presentation was also created that depicted the life of the senior and that included a developmental analysis. Paired sample *t*-tests revealed an increase in knowledge about older adults and aging following the program. Qualitative analysis of the students' personal reflections revealed several themes including intrapersonal development, and cognitive and emotional learning.

Lee [15] examined the reflective writing of 15 undergraduate college students who participated in a weekly, semester long nonfiction documentary film project based on the lives of older adults attending an adult day health center or living in a retirement community. Using narrative analysis, two raters uncovered five major themes which included “students' altruistic values emerge” and “students become advocates for challenging ageist stereotypes.” Students' altruism was reflected in statements that described wanting to honor the lives and capture the dignity of the elders' life stories. Others have examined the benefits of intergenerational programs that incorporate reminiscence or life review. However, these studies have focused on the effects on older adult participants alone [9].

While intergenerational programs that incorporate life review have been studied, older adults did not have the opportunity to generate their own written work nor did they have the opportunity to benefit from lifelong learning in the form of a writing workshop. Further, in some cases, only qualitative data was collected. A recent content analysis of studies incorporating intergenerational programs from the 1970s through the 2000s revealed the following limitations: limited use of theory and standardized measures, use of “soft” measures such as enjoyment, and assessment of only one generation of participants [4]. Mixed methods designs have been suggested to best capture the benefits of intergenerational programs [4]. Therefore to address this gap, the current study incorporated self-produced written autobiography as well as standardized measures, and the effects on both groups of participants were examined. Although details of psychosocial benefits for senior participants will be reported elsewhere (paper under review), a synopsis of results is included here. Erikson's theory of psychosocial development [19] and Levy's stereotype embodiment theory [20] provided the theoretical framework for this study and the researcher employed a mixed methods design. According to Erikson, the last two stages of human development include mentoring the next generation and reflecting on one's life experiences as a whole. The intervention described incorporates both components of adult development. As per Levy's stereotype embodiment theory, age stereotypes are internalized across the lifespan beginning in childhood. The environment, including mainstream culture, fuels the development of these stereotypes. The program in this study sought to combat the effects of mainstream culture by exposing students to positive images of community dwelling seniors. 

The purpose of the current study was to ascertain the feasibility of student recruitment, to evaluate the utility of chosen outcome measures, and to refine the intergenerational protocol for a larger scale study. However, preliminary results were also examined guided by the following research questions.

(1) What are the effects of an intergenerational exchange, based on discussions of autobiographical writings, on images of aging among student participants?

Using qualitative analysis,

(2) what are the experiences of students who have participated in an intergenerational exchange based on discussions of autobiographical writing produced by older adult participants?

## 2. Methodology

### 2.1. Design

The design for this feasibility study was a pretest, posttest design with a connected qualitative component. 

### 2.2. Participants

Older adult participants were recruited from one senior's residence in the Boston area (*N* = 6 participated in the writing workshop and *N* = 5 participated in both the writing workshop and intergenerational program). Individuals were 65 years of age or older and able to speak and write English and achieved a negative screen for probable dementia on the Mini-Cog. Students (*N* = 5) were recruited from the undergraduate and postbaccalaureate programs at Tufts University. 

### 2.3. Procedures

 The internal review board (IRB) application was submitted and approved. Recruitment of students included IRB approved flyers, an announcement on the student life website, and an information session for interested students which was held on campus. Students 18 years of age or older, who spoke English and who were interested in a career in the health sciences, were invited to participate. Following informed consent of interested participants, the baseline/demographic questionnaire was administered orally by the researcher. 

 The researcher then conducted the intervention using the “Share Your Life Story” writing workshop protocol [21], which takes place on a weekly basis for a total of eight weeks. The workshop provides the opportunity for seniors to write about their lives in a chronological sequence. Each week, participants are given writing prompts that relate to a specific decade of life. Participants have the opportunity to read their work aloud and receive positive feedback on their writing from the group leader and other group members. Only the older adult participants attended the writing workshop. Following the writing workshop, the “Image of Aging Scale” [22] was given to each student participant as a pretest measure. This questionnaire was self-administered and returned to the researcher before the start of the intergenerational exchange program.

The enhanced component of the workshop took place once a week for three weeks at the seniors' residence and was attended by both the older adult and student participants. The first session included self-introduction of all participants. Name plates were placed in front of each participant. Each session lasted approximately 90 minutes and included the opportunity for each older adult participant to read one piece of their work self-selected from their writings from the preceding eight-week workshop. Following each reading, a guided discussion took place between the older adults and students with regard to the contents of the writings. The ensuing discussions were facilitated by the researcher using preestablished questions/guidelines. Examples of discussion questions included “what are your reactions to the reading?,” “are there any aspects of the story you would like clarified?,” “did you learn anything new?,” and “can you identify with the story in any way?”

 Although one of the senior participants was more vocal than the others, the group leader ensured that each senior had the opportunity to share a story each week. Due to the time limit of 90 minutes for each workshop session, the group leader was able to redirect participants as needed by reminding them of the need to move on to the next participant. All students listened attentively. However, some commented about the stories and shared their own related experiences more than others.

Following the three-week enhanced component of the workshop, the Image of Aging Scale [22] was administered to the student participants and qualitative interviews were scheduled and completed. Seniors were asked to write about their experiences in the program. Following the qualitative interviews, a questionnaire, conducted orally, was used to generate feedback about the program and to seek suggestions on ways to improve it. 

### 2.4. Instrumentation

#### 2.4.1. Baseline Questionnaire for Student Participants

The baseline questionnaire developed by the researcher included data on age, ethnicity, education, and past experience/amount of contact with older adults. This information was used for the purpose of descriptive data on the student participants. 

#### 2.4.2. Image of Aging Scale

The Image of Aging Scale [22] is an age stereotype scale designed to assess both the positive and negative perceptions of older adults. Although it was validated among older adult participants, the tool was designed to be used with both the targets (older adults) and targeters (the young and older adults) of age stereotypes. The tool is made up of 18 items (individual words), nine reflecting positive stereotypes (e.g., “healthy”) and nine focused on negative stereotypes (e.g., “walks slowly”). Responders are asked to rate their perception on a seven-point scale ranging from “does not match my image” to “completely matches my image.” Each subscale of the tool is scored as a total out of 54. In a small sample of individuals (*N* = 20), test-retest reliability was demonstrated to be 0.92 for negative stereotypes and 0.79 for positive stereotypes over a one-week period. In a sample of 68 individuals Cronbach alphas were 0.84 for the positive stereotype component and 0.82 for the negative stereotype component. Further, the convergent validity of the positive age-stereotype component with the positive rating of the open-ended measure was 0.83. In summary, the Image of Aging scale is a reliable and valid measure of positive and negative images of aging.

#### 2.4.3. Semistructured Interview

Questions for the interview were developed by the researcher with the purpose of exploring the students' experiences in the program. Examples of questions include “Describe what the intergenerational experience was like for you” and “What kind of impact, if any, did it have on you?”

#### 2.4.4. Feedback Questionnaire on the Intergenerational Program

A questionnaire, developed by the researcher, sought feedback on the number and duration of sessions and asked for suggestions for improving the program. Both the seniors and student participants completed this questionnaire orally. Examples of questions included “How do you feel about the number of sessions (too many, too few or just right)?” and “What suggestions do you have for improving the intergenerational exchange experience?”

### 2.5. Data Analysis

Descriptive statistics were used to examine changes in pretest and posttest scores on the Image of Aging Scale. Due to the small sample size, inferential statistics were not used. SPSS version 20 was used for quantitative analysis. The qualitative data from the student interviews was analyzed using Colaizzi's [23] phenomenological analysis method and included: reading each participant's responses to acquire a feeling for them, extracting significant statements that pertain to the phenomena of interest (i.e., the students' experiences in the program), assigning meaning to each significant statement, and then organizing formulated meanings into clusters of themes. In a final step, themes were related back to the original interview transcriptions to ensure the themes did not propose anything that was not implied. Transcripts were re-read to ensure that no pertinent responses were unaccounted for in the themes and an audit trail was maintained.

## 3. Results

Although six seniors completed the writing workshop, only five participated in the intergenerational exchange portion of the program due to a scheduled vacation on the part of one participant. Five students initially signed up to participate. However, one dropped out due to a transportation issue. The IRB approved the recruitment of an additional student to fill the vacancy. 

Students ranged in age from 25 to 34. There were two males and three females. All had interacted with seniors in the past including contact with family members and elders in a work and/or volunteer setting. Amount of prior interaction with seniors varied from less than once a month to weekly. Four of the students held a bachelor's degree and one a master's degree. 

All senior participants had immigrated to the United States as adults and spoke other languages before English. All were Caucasian with the exception of one participant who was Asian. Four out of the five were women and they ranged in age from 78 to 87.

According to the results in Table 1, there was an increase in the positive images of aging and a decrease in the negative images of aging subscales following the program. Cohen's *d* = 0.2 represents a small effect, Cohen's *d* = 0.5 represents a medium effect, and Cohen's *d* = 0.8 represents a large effect [24]. Therefore, the effect sizes indicate a medium effect for negative images of aging and a large effect for positive images of aging. Although quantitative analysis suggests that the program had a positive impact on images of aging among student participants, the results do not provide any insight into how or why this occurred. Therefore, a connected qualitative component incorporating Colaizzi's [23] method of analysis was employed. One hundred and thirty two significant statements were extracted from transcribed audio recorded interviews. Arranging the formulated meanings into clusters resulted in six themes that included an enjoyable experience; insights into themselves and their own lives, exposure to life experiences different from one's own, personalization of historical events, changed perception of elders and the senior housing environment, and piqued interest in working with seniors.

### 3.1. An Enjoyable Experience

All student participants found the program to be a positive, enjoyable experience. Responding quickly and spontaneously, students used words such as “interesting,” “enlightening,” and “fascinating” to describe their overall experience in the program. For some students it was a welcome break from their hectic school schedules. The experience was described as “relaxing” and “calming.” For others, the experience was inspirational, especially given that many of the senior participants had lived through World War II and shared some of these experiences. One student stated: “You aspire to be able to handle anything that comes your way after hearing people who survived that.” Given that four of the five senior participants were retired professional women, it was not surprising that inspiration came in other forms. One female student stated: “It was all very girl power to me.” A male premed student was inspired by the active, engaged nature of the participants and found the behavior among participants to be a sharp contrast to what he had experienced with his own older adult relatives. He stated: “I would like to emulate that sort of continuous engagement” and “They were inspiring because I think I would like to make lifelong learning a part of my life.”

### 3.2. Insights into Themselves and Their Own Lives

The experience caused students to reflect on their own lives in a variety of ways. One student discovered an interest in learning more about personal accounts of historical events: “I feel like I want to go and read a little more of biographical accounts of individuals.” It caused this same student to reflect on her communication skills: “sometimes, whenever I am talking with someone, if I do not quite understand what they are saying sometimes, I just pretend to understand…that is something that came to mind while we were interacting.” For others, it resulted in some soul searching with regard to their views of elders. One student stated: “It sort of forced me to look at my own conceptions and stereotypes.” It provided one female student with a new perspective on her life stressors and caused another to reflect on her family care-giving preferences. The second student stated: “It was kind of after this that I decided that my parents will always, whatever happens, they will always live with me.”

### 3.3. Exposure to Life Experiences Different from One's Own

Several students described how their own life experiences were very different and much less dramatic than those of the seniors. For example, one stated: “[I]t was very different obviously from my own upbringing.” Another noted: “[T]he events that they lived through, namely World War II, communism, immigrating to the United States, [are] things that I never lived through.” The program provided student participants with cultural insights and opportunities to learn about the immigrant experience as reflected in the following statement: “I definitely learned that being an immigrant must have been a difficult experience in this country.” One student reflected on a story he had heard about the close relationship one participant had formed with the family for whom she worked and described it as “an interesting view into how people who have immigrated here have made different sorts of families.” 

### 3.4. Personalization of Historical Events

For many of the students it provided a personal account of events they had only read about. A comment from a male student illustrates this point vividly: “…but then hearing individual stories about how those large muscle movements affected that individual fleshes it out.” One student, who had travelled to South America, reflected on the opportunity to hear about a participant's experiences as a foreign worker in Uruguay during the 1950s' “…[J]ust hearing why he went there and what he did while he was there was kind of putting a personal story to that fact.” It provided students with a personal account of life in Europe during World War II. One student stated: “That trajectory of her life as a refugee was very interesting and I never heard what that journey was really like.” According to another: “It gave me a weird picture of a war torn area that I did not have before.”

### 3.5. Changed Perception of Elders and the Senior Housing Environment

Some students reported that the experience changed their perceptions of seniors in general, and for others, it altered their perceptions of elders who reside in senior residences. One student remarked: “I did not see people that matched the view that I had.” Another stated: “I was not expecting to see people who were as healthy or as vibrant as they were.” Hearing stories and engaging with seniors who had participated in the creative art of writing appeared to provide a unique opportunity: “It reinforced the idea that elderly people can still have that joy of life, that ability to create something and be creative and put their life stories into that creation.” According to another student “I never once in my life thought that I would see…that people as they get older they would pick up hobbies and things that they enjoy and learn new things.” The group format also provided opportunities for students to see seniors interacting with one another: “I really enjoyed watching the interactions, but especially between seniors… It was so interesting to see people encouraging one another.” 

 Some students felt that the experience was humanizing. One student stated: “It was interesting to see them being, I do not know, just like everyone else.” Another student, who had interacted with seniors in a hospital setting where she felt there was little opportunity to get to know patients, remarked “… to take the time to get to know people…to put a background and a real life to the person in front of me.” Others reported that it provided concrete examples of individuals who are “aging well.” For example, one female student reflected on the level of activity she observed among participants: “[T]hey are busy. I did not realize that seniors were that busy.” One male student commented: “It was nice to see people able to recall those moments so many years later.” Another stated: “These people are constantly learning new things” and “there was not a visible decline.” The same student also commented: “It definitely made me, like I said before, fully aware that lifelong learning and engagement is really an important piece of aging well.” Several students used the word “resilient” to describe the senior participants. And one student stated: “There are a lot of people who have overcome a lot of really awful things that are still positive, still active, still energetic.” 

### 3.6. Piqued Interest in Working with Seniors

Changed perceptions were translated into an enhanced interest in working with seniors. For two students the experience sparked their interest in future volunteer work. One of the female students stated: “One of my colleagues, her partner runs an assisted living….It is kind of a Big Brother/Big Sister thing except it is for an elderly person…I should go and do that.” For some student participants it enhanced an existing interest in working with seniors in a professional capacity. One male student reported: “I think even before this I had an interest in aging… it has been in addition to what I have experienced in the past that affected what my trajectory might be.” For others, it sparked an interest where there was none before. One student explained: “I am not so quick to write it off anymore…It is definitely something I would consider.” One female student's past interactions with elders included a negative experience with a family member who had poor health and who was verbally abusive. She stated: “I always thought working with seniors would be depressing…If I was treating the health of people who are like these folks it wouldn't be…It definitely changed the way I thought it would be to work with seniors in a medical capacity.” Students' responses highlight how the change in interest level may have come about through this experience. One remarked: “It has piqued my interest because there are a lot of experiences to be mined.” Another student stated: “They are interesting people who have had interesting lives” and “I think there is a lot of information and wisdom we can get from older people.”

Results also suggest benefits for senior participants. Although there was no incremental improvement in geriatric depression scale (GDS) scores following the intergenerational program (when compared to GDS scores after the writing workshop), an analysis of seniors' written responses describing their experiences in the intergeneration portion of the program revealed therapeutic benefits. Written responses were analyzed using Linguistic Inquiry and Word Count Software (LIWC), a text analysis software program designed to calculate the extent to which people use certain categories of words. Examples of word categories include affective processes (e.g., positive and negative emotions), cognitive processes (e.g., insight and causation), and perceptual processes (e.g., hear and see). Seniors written responses revealed six times as many words indicating positive emotions than negative emotions and a large percentage of words indicating cognitive processes. Detailed findings related to the senior participants are reported elsewhere (under review). 

## 4. Discussion

Using a mixed method feasibility study, the researcher sought to (1) explore recruitment potential and appropriateness of chosen measures and refine the protocol for the intergenerational portion of the intervention; (2) examine preliminary results for the impact of an intergenerational program based on the autobiographical writings of seniors on images of aging among student participants; (3) explore the experience of students who participated in the program; and (4) examine enhanced therapeutic benefit of the intergenerational program as compared to the original protocol. 

Quantitative results reveal that the program had a positive impact on images of aging for the five student participants. This included a decrease in negative images of elders and an increase in positive images of elders. Noteworthy is that the effect size for changes in positive images of aging is much larger than that for negative images of aging. Therefore, negative age stereotypes may be more difficult to change through this type of intervention. 

 Qualitative results help to explain the hows and whys of the quantitative findings. Student participants found the experience to be enjoyable and the program resulted in new learning about themselves and others. It also exposed students to active, engaged seniors who are “aging well.” Demonstration of cognitive ability was made evident in the recalling and writing of life stories. Further, the program provided the unique opportunity to engage with seniors through an exchange based on their creative works. These combined experiences may in part explain the positive changes in images of aging. 

The results of the study are consistent with past findings with regard to changes in attitudes and images of elders following intergenerational programs [7, 15]. Lee's [15] study had a similar finding with regard to the emergence of students' altruistic values following participation in an intergenerational exchange based on life review. In Lee's study, altruism was reflected in the desire to preserve the dignity and honor of elders through a video representation of their lives. In the current study, students expressed altruism through their desire to help seniors in a volunteer and/or professional capacity. Consistent with Zucchero's [18] findings, students were inspired by the senior participants and learned from them. They also gained new insights into themselves. According to the results of this study, intergenerational programming based on the content of the autobiographical writings of seniors may help to combat the effects of the environmental exposures (e.g., mainstream culture) described in Levy's stereotype embodiment theory [20]. 

Outcomes for seniors, including positive emotions and cognitive processes, are also consistent with past findings. Zucchero [18], who studied a life review program conducted by undergraduate psychology students, uncovered the following themes from interviews with seniors: positive emotion or experience, a shift in their own perspective, and a changed view of young adults.

 Limitations of this feasibility study include the small convenience sample and the use of only one coder for qualitative analysis. Some students may have chosen to participate in the study due to a preexisting interest in working with elders. Larger experimental studies with multiple coders for qualitative analysis are warranted in the future. Long-term effects of the program on student's perceptions and attitudes are unclear. A longitudinal study could help determine the need for repeated exposures over time. 

Feedback from both student and senior participants was helpful in refining the protocol for the intergenerational exchange program. Going forward, the duration of the intergenerational program will be increased to four sessions as participants felt that three sessions were too few. Clearer initial guidelines regarding student participation will be included in the protocol as well as a stronger focus on discussion questions that encourage students to share their own related experiences. Strategies for communicating with individuals who have hearing loss will also be added to the student orientation. At the completion of the program, students will be asked to write a brief summary about their experiences in the program and this information will then be shared orally with the seniors. This will provide a mechanism for seniors to receive feedback on how much the students benefitted from hearing their life stories. 

While a larger scale study is called for to evaluate the effectiveness of the program, preliminary results are positive. Given the projected shortage of health care professionals who specialize in geriatrics, programs that target perceptions of and interest in working with older adults will be increasingly important going forward. This innovative program warrants further investigation as an intervention that targets attitudes and interests of students who intend to pursue a career in the health sciences. Moreover, nonhealth science students could also benefit from the program to provide them with first-hand accounts of historical events, to promote civic engagement with elders in the future, and to challenge their perceptions of aging. Positive perceptions of older adults and aging not only promote social justice, but also play a key role in future health outcomes [25].

## Figures and Tables

**Table 1:** Descriptive statistics.

	Pretest M (S.D)	Posttest M (S.D)	Change score M (S.D)	Effect size (*d*)
Positive image subscale	34.00 (5.20)	39.80 (6.69)	5.80 (1.79)	3.24
Negative image subscale	29.40 (4.16)	22.40 (10.09)	−7.00 (10.15)	0.69

Subscales from: Image of Aging Scale—Levy et al., 2004 [22].
